# Using linked healthcare data to create a randomised controlled trial: the rapid trial (reducing antibiotic prescribing in dentistry)

**DOI:** 10.1186/1745-6215-14-S1-O16

**Published:** 2013-11-29

**Authors:** Paula Elouafkaoui, Andrew Elders, Jan Clarkson, Eilidh Duncan, Maria Prior, Craig Ramsay, Linda Young

**Affiliations:** 1 NHS Education for Scotland, Dundee, UK; 2 University of Dundee, Dundee, UK; 3 Health Services Research Unit, University of Aberdeen, Aberdeen, UK

## 

We report the design of a three-arm RCT to compare the effectiveness of enhanced audit and feedback strategies for the translation into practice of published guidance on antibiotic prescribing in dentistry. The trial uses linked healthcare data from administrative datasets (dental workforce data linked with claims for treatment provision and pharmacy data) in five aspects of the trial to:

**1. identify participants:** all currently practicing General Dental Practitioners (GDPs) in Scotland.

**2. apply inclusion/exclusion criteria**; based on dental practice contract status and a minimum level of recent treatment provision.

**3. carry out stratified randomisation**; all eligible dental practices in Scotland were simultaneously randomised at baseline to current audit practice or to one of two audit and feedback interventions. Randomisation was stratified by single-handed/multi-handed practices. Intervention practices were further randomised using a factorial design.

**4. generate the trial intervention**; individualised graphical feedback on antibiotic prescribing. The initial feedback report contained 14 months retrospective antibiotic prescribing data.

**5. analyse trial outcomes**; the primary outcome is the total antibiotic prescribing rate per 100 courses of treatment over the year following the delivery of the baseline intervention.

In describing the design of this study, we demonstrate that linked administrative datasets have the potential to be used efficiently and effectively across all stages of an RCT. We also discuss the various challenges and limitations that such an approach presents.

